# Academy of Stomatology

**Published:** 1898-08

**Authors:** 

**Affiliations:** Academy of Stomatology


### ACADEMY OF STOMATOLOGY.

A REGULAR meeting of the Academy of Stomatology of Philadelphia was held at the rooms of the Academy, 1731 Chestnut Street, February 22, 1898, Dr. Edwin T. Darby in the chair.

A paper, entitled "Physical Conditions that determine Choice of Filling-Materials," was read by Dr. S. B. Luckie.

(For Dr. Luckie's paper, see page 503.)

### DISCUSSION.

*Dr. H. H. Burchard.*—There are four elements, as I view it, to consider in the subject of Dr. Luckie's essay. The first of these is the filling-material. Filling-materials are, in reality, therapeutic agents, and they should be applied with a full regard to the chemical and physical properties of the agent; that is to say, their application is based on their physical and chemical properties. For instance, zinc phosphate has certain physical and chemical properties, which, when brought in contact with organic tissue, exhibits a certain reaction against this tissue, and so with gutta-percha, amalgam, etc., that is the first element to consider. An exhaustive knowledge of this is the *a priori* basis upon which the choice of filling-material in any given case depends. The second element is the question of the condition to which these agents are to be applied,—that is to say, the dental condition which we ought to treat with these therapeutic agents, that involves the condition of the tooth itself, what condition the tooth may be in, any amount of loss of tissue, the condition of remaining tissue, and so on.

The essayist took up what is an extremely important matter, and one, too, perhaps, to which not sufficient attention has been directed,—that is, the nervous reaction of the patient after operation, the question of shock. This is a matter which varies with the individual, and, as he has well said, it may be as much psychical as physical. However, the effects in both cases may be alike severe. Granting the skill of the operator, and given a clear understanding of the condition of the tooth, an exhaustive knowledge of therapeutic agents, and the ability of the patient to withstand any necessary operation, the question arises, With what material can he best accomplish a definite end,—that is, of course, the sealing of the cavity?

To discuss the subject exhaustively would mean the running over of all the literature of the plastic fillings and a general review of dental pathology. But there is one point in his quotations from Dr. Black that I want to comment upon, and that is the question of density and the amount of calcium salts contained in the tooth. These are not necessarily associated with hardness of the tooth. The dentine may be hard or it may not, while exhibiting an unusual percentage of calcium salts, and it may be hard and not notably dense.

These factors are not necessarily related to one another; but if I begin with it, it means a review of Black's work, and that would be two weeks' work.

*Dr. R. Huey.*—Mr. President, this is a subject that is intensely interesting to all of us whose lives are devoted to the saving of teeth. I do not know that there is but one subject that I have ever thought more of in connection with operative dentistry, that interests me more than this we are talking about to-night. There is, I think, a line that should be drawn in regard to the use of plastic materials. I am very jealous for myself, for my time, and for my work, and I do not like to put my best work into mouths where there is a very decided probability that this may not last. I would draw my line at the condition of the mouth, and consider carefully the amount of fermentation, and the care which the patient bestows upon the teeth and their surroundings. I think that is really more important than the density or the calcification of tooth-structure. My experience has been that it does not recur just where it is supposed to recur. We are told that the weakest point is the cervix, and we expect decay to recur at that point. My experience has been that it usually takes place at either the labial or the lingual side of the cervix, unless the filling has extended out so far that the tooth will be thoroughly cleansed during mastication or brushing. It is almost impossible to get the patients to push silk back to the angle of the tooth and draw it forward, cleansing that point, which is to me the most dangerous. For that reason I do not like to put permanent work in a mouth where I am satisfied the patient will not take care of it. What I consider most is not the amount of calcification, but the education of the patient. In consequence of these facts I often carry a patient along as well as I can with plastics until the time comes when other work can be done.

*Dr. H. C. Register.*—The subject of the selection of filling-material is one resting mainly with the individual operator and with the individual case at the time of operating. I believe that there are a great many patients whose teeth would be lost if we undertook to operate on them at one time with a material like gold, while, if it was postponed until the patient was in physical condition to meet the operation, success would be perfect. I have had several instances where the most trivial operations have been productive of quite serious results. I was once operating for my brother, and doing a very simple thing, when he fainted. I had hardly touched him, and yet it was because he apprehended being hurt. I have had several cases where patients, simply from fear, would almost faint. The idea suggested itself to me a few years ago to use some little attractions in my office to divert attention, and for that purpose I placed near the operating-chair a little aquarium. I even built a bird-box, and put it outside on the lattice for the sparrows of Philadelphia. I cannot but believe that success in filling teeth and the selection of materials to fill them depend very much upon the mental and physical condition of the patient.

*Dr. F. Gardiner.*—It is very difficult, indeed, to lay down a rule for every one to follow. Each man has to be governed by what he can do in a certain case; and in regard to filling proximal surfaces, particularly with gold, I think it largely depends upon the principle which is carried out. I should want very strong teeth, indeed, before deciding to fill a small cavity between bicuspids and molars with gold, because it is very difficult to change the conditions. The same conditions remain after a tooth is filled that existed before, and that caused the tooth to decay. The probability is that decay will recur in a comparatively short time. In larger cavities, I think, we can venture to fill with gold very much more freely than we could in smaller cavities, and expect a permanent result. We have an opportunity of changing the conditions and of extending the cavity, so as to obtain self-cleansing surfaces, and to separate the tooth-substance absolutely from contact with an adjoining tooth. That of itself is a great hygienic measure. Each man should be the judge of what he can do himself rather than what others can do under like conditions, for more depends on that than upon the structure of the tooth.

*Dr. James Truman.*—The question that the essayist has introduced in regard to the effect produced on the mind as well as the body by operations seems to me one of vital importance. Some sixteen years ago I wrote for the Odontological Society an article on "Shock," which was received rather dubiously. In my opinion it is not only a physical depression to which our patients are liable, but also a psychical one, and that is probably as important as the physical. The effect of shock upon the vasomotor system of nerves produces an anæmic condition. I have seen it so often and so markedly produced in the clinics of the institutions in which I have had the honor of being present, that I have come to regard it as somewhat alarming. I believe that few persons can sit as long as seven hours in the dental chair and not be brought into a depressed condition, tending to serious injury, and I hold, therefore, that any dentist so operating is legally liable. Two hours in the chair is all that is just to any patient, and not that without some study of the peculiarities of the individual. I am satisfied that a vast deal of harm is done in this direction, and that the essayist did well to call especial attention to it.

In regard to Dr. Black's idea of density, he holds that it is specific gravity, which we all understand to be density; but the dentist does not understand it that way. When a dentist begins operating on a tooth, he expects to decide the density of that particular tooth by his excavator, and if it cuts hard he feels that he can introduce a filling that will be satisfactory to both himself and his patient. If it presents that well-known soft appearance, he is aware that he cannot do that kind of work, and therefore must use something that has a therapeutic value, and wait until such time as the tooth becomes more dense. It seems to me that the ideas that have been promulgated in regard to this are wrong from a clinical stand-point. They have led many men to suppose that they could treat children's teeth—young womens' teeth, along from fourteen to fifteen—with any kind of material; for Dr. Black has said that all teeth are sufficiently dense to be filled with metal. I am on record and hold to the opinion that children's teeth should not be filled with gold except under exceptional conditions. If we are to adopt theoretical ideas, such as Black and others have in regard to this matter, it seems to me that it will upset the experience of more than one generation of dentists. It has come down to the practical point that some teeth cannot be filled with metal. No matter how much we like it, or prefer to use gold or amalgam, or any of the other materials that we have, we must resort to the less resistant plastics for the purpose of saving these teeth, until the organic bases of the teeth increase their density.

*Dr. O. E. Inglis.*—I would like to put a little more emphasis on what Dr. Huey said in regard to the environments of the tooth. I think it is largely that upon which the choice of filling-material is dependent. I certainly, for my part, have quite a number of patients in whom nervous prostration, indigestion, or other physical troubles have superinduced oral conditions favorable to caries.

In regard to the cleansing of the teeth, we all know that if teeth get into a habit of decay, they need thereafter very careful attention. The slightest projection or depression of filling-materials invites caries at the margins of the fillings. It becomes impossible at times to cleanse the mouth thoroughly. Under adverse circumstances we have to use the softer materials. Let us take, for example, the buccal surfaces of the third molars, and especially the buccal surfaces which are superficially decalcified over the entire surface. When a patient is nervous and bears operations very poorly, it is sometimes impossible to carry the margins of the cavity to a satisfactory completion. When that is the case, if gutta-percha and copper amalgam are used and renewed at reasonable intervals, these teeth are very frequently slow in decaying, especially if the health of the patient is restored.

*Dr. C. E. Jeffries.*—The subject of the selection of material for any given case is one of the most important in our calling. All of the list can be gone over to advantage in individual cases, even in the same tooth. The same material does not fit every case; we can use two, three, and sometimes four materials to advantage, instead of using one alone. Dr. Black has been quoted this evening as saying that he could put gold where he could put any metal to advantage in young or old. Now, he also says in some of his other writings that any kind of metal filling of any considerable size placed in a tooth will modify more or less the pulp and endanger its safety, if not in a few months or a year, then in ten years. It is very rarely the case that it does not; and it would seem to me that a man with such a general knowledge and thorough powers of investigation is making a very unwise statement to say that gold can be put in any tooth where metal can possibly be used. We know that gold is the best possible conductor, and therefore the worst material to put in a delicate tooth, regardless of age; but more particularly for young persons. I certainly cannot harmonize these statements coming from Dr. Black, and I do not understand his line of argument that in one case he would favor gold in all places where metal can be put, while he says in another that any metal fillings of any considerable size will modify the condition of the pulp, almost invariably to its disadvantage.

I think we cannot be too particular in the selection of our materials for filling, especially in the young. I use myself a great deal of gutta-percha, and I have the utmost satisfaction in its use for young patients, as well as for some of the older ones. It is one of the most valuable materials that we have, and even if it does wear away, it is easily renewed. It is very seldom that we find evidence of decay about a gutta-percha filling, even when half worn away. Some of the cements do good service, while the much-abused amalgam is not used in the majority of cases in a way to bring about the best results from its use. Upon proximal surfaces it is quite the rule to leave the fillings unfinished, a condition which invites decay in the future; whereas, if they were finished as gold is, they would do better than gold. I do not think that will be disputed. So, to my mind, selection is a very important matter, and one of which the gold *fiends* are not sufficiently cognizant.

*Dr. Louis Jack.*—In nearly every instance when patients have come to me, excited over the approach of an operation, even if not threatening to be painful, I have found the use of aqua camphora of exceeding value, and several patients ask me for it—after having found the beneficial results obtained—before the operation is commenced. In some instances I am satisfied that the condition is induced by the momentary failure of respiration, and the aeration of the blood not being sufficiently sustained tends to increase the physical and nervous depression. You will observe in the consideration of the effects of the action of camphore tincture, or aqua camphora, that it tends to lessen nervous perturbation. The manner in which I use it is to allow the patient to gargle and rinse the mouth, and in marked cases I allow them to take about a drachm internally.

*Dr. O. E. Inglis.*—The use of chloral hydrate, under such circumstances, works nicely; five grains administered to the patient before the operation rather calms the nervous system and brings about a condition which enables one to operate very well.

*Dr. Wm. Trueman.*—The paper to which we have listened to-night is one of those of which I wish we could have more; it is carefully and thoughtfully written and thoroughly practical from beginning to end. As the doctor suggests, in the case referred to, the gold filling comes back to us and we find discoloration about the edges and considerable organic matter which must be removed.

We all recognize that there are some classes of teeth that we must treat very gently; the softest fillings probably preserve them best. I know what it is to sit in a dentist's chair from eight o'clock in the morning to five in the afternoon. I know what it is to spend ninety-six hours in the chair in a week. And what was the result? Dr. Webb did as well for my teeth as any man could do, but in about three years one of the four teeth which I had filled broke down; in about seven years more another followed. Dr. Webb seemed to think that a solid gold filling would improve frail tooth-structure. It proved otherwise. In the case of many gold fillings put in with all possible care, after a lapse of a dozen years (perhaps not so long) there is a wearing down of the tooth-tissue, and the gold, not wearing so fast, breaks away. I think that while rules for our guidance may be laid down, all these must at times be laid aside and our own practical experience be made the judge.

*Dr. R. Huey.*—I believe all of us agree that the teeth ought to be well calcified before a gold filling is inserted. I want the gentlemen either to condemn the practice or to confess that there are times when they feel that a gold filling ought to be inserted before this calcification has taken place. I have one pet habit of inserting a gold filling upon the mesial surface of a first molar before the second bicuspid comes in. I like very much to get a gold filling in this situation, because I can do it more thoroughly and easily and in a shorter time, yet sometimes my conscience pricks me if I have recurrence of decay at this point for having made that operation. In a great many cases the operation has partially succeeded better than if I had wedged the teeth apart and filled it. I would like to hear what some of these gentlemen think of this practice and whether they follow it.

*Dr. J. A. Woodward.*—That also is a weakness of mine. I prefer to fill the mesial surface when the temporary molar is lost and before the second bicuspid comes into place, but I sacrifice the enamel and dentine until the edges of the filling will be free from contact except at the point where the teeth touch. With these fillings I have been very successful.

Dr. Luckie quoted from Dr. Black that the density of the dentine has very little to do with determining whether the tooth shall be filled with metal or not. Some years ago it was my fortune to be associated with a very skilful operator who would fill the first molars on the proximal surface with gold even when he had to push the gum back with a ball of cotton. Those fillings have always done well. I do not know that I have seen any recurrence of decay about such fillings. This fact led me to doubt whether the density of the tooth had much to do with the durability of metal fillings. I think there is more in the manner in which a tooth is filled.

I use gutta-percha largely on the occlusal surfaces of the molars and bicuspids of children, but as soon as possible I change to gold, without regard to age, and I do not think the surroundings have much to do with it. I think that gold well introduced, if not broken up or marred by mallets or improper instruments, makes as good a filling as any other material. It is better than amalgam, and such fillings, when properly introduced, have resulted satisfactorily.

With reference to decay about proximal fillings, I find that oftentimes it will start at a distance from my filling, and if the examination be closely made, a sound portion of enamel will be found between the filling and the new centre of caries. We may thus blame ourselves for making defective fillings while really decay has occurred at another spot.

*Dr. C. E. Jeffries.*—I should like to know if those gold fillings were not entirely of non-cohesive gold and placed in with smooth points.

*Dr. Woodward.*—They were of cohesive gold and placed in with serrated points.

*Dr. James Truman.*—The idea of placing fillings of gold against the soft structure of certain teeth seems to me altogether erroneous. I cannot understand how such fillings can be successful. In my experience the conductivity of the gold permits thermal changes to eventually produce congestion and death of the pulp.

I should fill the mesial surfaces of the first molar with tin-foil and wait until that increase of density, of which Dr. Black speaks as coming in the course of years, takes place. If used upon the cohesive principle, tin, which is a very poor conductor of heat and cold, is the best metal for use where gold is contraindicated. I do not think metal is always required. For twelve years I worked in the Institution for the Blind in this city and used nothing but tin, rarely placing in a gold filling, and many of these teeth are well preserved to-day. That is a very good test when we consider that the patients were children. We do not appreciate tin sufficiently.

*Dr. Woodward.*—What I said was based on experience. Theoretically I know there is much objection to gold in young teeth. Large cavities are not usually found in young teeth, consequently you can fill them with gold.

Speaking of Dr. Huey's anterior surfaces of the motars, I would not want tin there, but gold, something that would retain its contour.

*Dr. Louis Jack.*—I have been filling first molars with gold, but would not do so if the cavity was deep. If I did I would line the bottom of the cavity with a non-conductor.

*Dr. O. E. Inglis.*—I can see no reason why a cavity on the mesial surface of a first molar cannot be filled with gold. If the fillings are contoured, as Dr. Woodward states, with self-cleansing surfaces upon the buccal and lingual side, the only recurrence of decay would be at the cervical margins. If caries does recur, it can be repaired with amalgam, gutta-percha, or tin. As far as the masticating surfaces are concerned, I think it is a common observation that well placed fillings which are resistant of mechanical and chemical forces will last. If gold is used, they will probably last as well as when made with anything else, perhaps better.

*Dr. R. Huey.*—Just another factor. Admitting that we can make a lasting filling with gold in the young tooth, is it fair to the young patient or to the dentist? I find in my practice that a young child always has a certain nervous dread, and if I fill that child's tooth with gold it will have a horror of dentistry for which I am to blame, but if I carry that child along with plastics until the thirteenth or fourteenth year it has no dread of dentisty. I have not hurt that child unnecessarily and I have saved the tooth.

We very frequently have cavities in this situation that are not more than a thirty-second of an inch in diameter,—after everything is removed. If the child is in the hands of a careful dentist, it is very rarely that these cavities reach any size. Some one is to blame if they do. We often find a very slight infection of the enamel on that surface. The question is, Should we fill or let it alone?

*Dr. Woodward.*—In that case I should polish it out without destroying its contour. If I could not do that, and it was slight, I would let it alone.

*Dr. R. Huey.*—How about the cavity in the second bicuspid later?

*Dr. Woodward.*—I would take care of that.

*Dr. S. B. Luckie.*—I do not know that there is much that I can add. The subject is not yet exhausted. The intent of the paper was to show that the basis of selection lies in the physical condition of the patient and also the physical condition of the tooth; and at the same time, I did not ignore other conditions that I considered of secondary importance, as, for instance, the hygienic conditions of the mouth. Another object of the paper was to provoke a discussion upon Dr. Black's conclusions. On that point I am somewhat disappointed. There are times when the physical condition of the patient indicates a gold filling or other prolonged operation, and yet the condition of the tooth counteracts it, and *vice versa.* A lady for whom I operated carefully for eighteen years, never exhibited the least sign of exhaustion, though being in the chair many times as long as two hours at a time, until this winter, when two teeth that had been filled with plastics were required to be refilled. She made the appointment and I reserved an hour and a half to fill them. I had the teeth filled in a little less than that time. I noticed she was somewhat fatigued when getting out of the chair. She had to go home and go to bed, she was so exhausted. That was a case where the physical condition of the teeth demonstrated that they could be filled with gold and yet the physical condition of the patient was not favorable to it. To-day I finished a lady's mouth who nearly always has had her teeth filled with gold. Two years ago she came to me after having typhoid fever, and for the first time I told her that I would not fill her teeth with gold but with amalgam. I told her that her teeth required more filling than at any time during the period I had her under my care,—probably fifteen years,—and yet a number of fillings showed a degeneration of the structure of the teeth around the fillings. I can take the edge of an excavator and chip the tooth away from the filling, a number being in that condition. I refilled a number of them, requiring large fillings; all were gold except one, the lower third molar. There are times when gold should not be used, when we should return to plastics, and expect nature to assist us in getting them into condition for permanent work, and yet young children's teeth are often in such a condition that gold can be used quite as well as in the teeth of older people.

The matter of filling the first permanent molar, as suggested by Dr. Huey, depends very largely upon the judgment of the operator and his ability to manage the patient at the time. I am very much averse to filling the teeth of children with gold; I prefer to treat them as gently as possible and make it as easy for them to return, when necessary, as I can.

Otto E. Inglis,
*Editor Academy of Stomatology.*

